# Development of a LC-MS/MS method using stable isotope dilution for the quantification of individual B_6_ vitamers in fruits, vegetables, and cereals

**DOI:** 10.1007/s00216-020-02857-5

**Published:** 2020-08-14

**Authors:** Thomas Bachmann, Andrea Maurer, Michael Rychlik

**Affiliations:** Chair of Analytical Food Chemistry, Technical University of Munich, Maximus-von-Imhof-Forum 2, 85354 Freising, Germany

**Keywords:** Food, LC-MS/MS, Stable isotope dilution assay, Pyridoxine, Pyridoxal, Pyridoxamine

## Abstract

Vitamin B_6_ comprises an important set of molecules tightly interwoven with the human amino acid, fatty acid, and carbohydrate metabolism. Analytical methods striving for the quantification of individual B_6_ vitamers so far mostly rely on methods based on HPLC in combination with fluorescence detection, but their application encounters multiple difficulties due to the chemical divergence of the single vitamers. The present study describes the development of a method based on LC-MS/MS and stable isotope dilution assay (SIDA) for the simultaneous quantification of five vitamers (PN, PL, PM, PMP, and PNG) of the B_6_ group in food samples. [^13^C_3_]-PN, [^13^C_3_]-PL, and [^13^C_6_]-PNG were applied as internal standards for the analysis of PN, PL, and PNG. PM and PMP were quantified via matrix-matched calibration referring to [^13^C_3_]-PN. The developed method was validated using starch matrix. The limits of detection and quantification ranged from 0.0028 to 0.02 mg/kg and from 0.0085 to 0.059 mg/kg, respectively, for all analytes. Calculated recoveries varied from 92 to 111%. Intra-injection precisions ranged from 0 to 9%, inter-day precisions from 4 to 10%, and intra-day precisions from 4 to 10%. A total of 14 plant-based food samples *including* fruits, vegetables, and cereals were examined for their content of vitamin B_6_ using the validated method. Furthermore, the first quantitation of PNG without enzymatic steps or divergent internal standards was undertaken utilizing LC-MS/MS and SIDA.

**Electronic supplementary material:**

The online version of this article (10.1007/s00216-020-02857-5) contains supplementary material, which is available to authorized users.

## Introduction

**Fig. 1:**
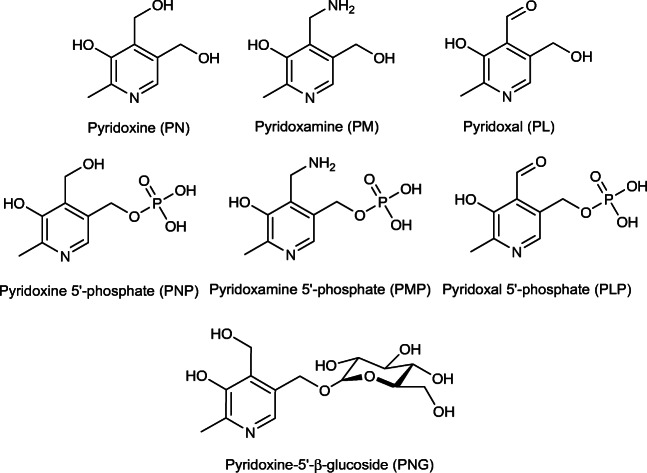
The vitamin B_6_ group unites substrates sharing a 2-methyl-3-hydroxypyridine structure

The group of vitamin B_6_ encompasses several water-soluble, essential, yet in vivo inter-convertible, vitamers, namely pyridoxine (PN), pyridoxal (PL), pyridoxamine (PM), and their respective phosphorylated compounds pyridoxine 5′-phosphate (PNP), pyridoxal 5′-phosphate (PLP), and pyridoxamine 5′-phosphate (PMP) [1]. In plants, the glycosylated derivative pyridoxine-5′-*β*-d-glucoside (5′-*β*-PNG) accounts for the major fraction of the total vitamin B_6_ content, additionally (Fig. 1) [2–4].

Vitamin B_6_ plays an important role in the human metabolism where it exhibits co-enzymatic characteristics in its metabolically active form, PLP, and is involved in an astonishing variety of over 150 biochemical reactions—an estimated 4% of all enzyme activities in the human metabolism show PLP dependency—within the amino acid, fatty acid, and carbohydrate metabolism [5, 6]. The diverse spectrum of enzymes furthermore increasingly awakens interest as potential targets in the field of drug development [7]. Additionally, vitamin B_6_ has been correlated to a number of physiological disorders, e.g., cardiovascular diseases [8], cancer development [9], oxidative stress [10], chromosomal instability [11], and inflammation [12].

The multifarious number of studies undertaken throughout medical subjects also illuminated analytical problems regarding this group of molecules. More precisely, the simultaneous quantification of the single vitamers presents a challenge to the analytical world to this day. The number and instability of the molecules, their high polarity, the similarity in structure, and connection to the examined matrices outline just some of the factors enriching the aggravating complexity of a specific individual determination [13–16]. Hence, a numerous variety of methods using several principles, e.g., microbiology [17–19], electrochemistry [20], spectrometry [21, 22], and chromatography [23–34], has been developed to quantify vitamin B_6_, either as part of a multivitamin method or in a selective screening of the B_6_ group. Many traditional methods pursue the detection of specifically selected vitamers or have to rely on the enzymatic transformation into PN beforehand [25, 32, 35]. Although the determination of individual B_6_ vitamers demands a higher analytical complexity, the benefits granted through this effort are of enormous nature to nutritional and medical fields. This is reflected in the aspect that, while the human body not being equipped with an ability to synthesize vitamin B_6_ itself, it possesses a fine-tuned mechanism of transforming individual vitamers of B_6_ into each other. The grave importance of this process with regard to the absorption, transport, and bioavailability inside the body is mirrored by the divergent utilization of the different forms of B_6_. While PLP and PL are the major forms present in the blood stream, phosphorylation and dephosphorylation present necessary steps for the transport and metabolic trapping processes inside the brain cells and choroid plexus. Hence, a method giving access to a more wide-ranging information via determination of individual quantities of the different forms of B_6_ compared with methods using multivitamin determination or transformation into one vitamer allows a deeper insight into the nutritional value of different foods and more detailed data when working with biological samples.

The increasing technological progress and involvement of liquid chromatography (LC) systems in the analytical field, mostly in combination with fluorescence detection due to the inherent sensitivity for B_6_, immensely facilitated aspects like simultaneous tracing of multiple substrates [36]. Nevertheless, issues like low fluorescence quantum yield, matrix interferences leading to signal-intensity manipulation, necessary pre-/post-column derivatization, and insufficient separation of the vitamers on the solid phase remained. Recent developments show interest in the application of systems based on liquid chromatography coupled to tandem mass spectrometry (LC-MS/MS). The involvement of isotopically labeled internal standards compensates for losses during sample workup and thus minimizes systematic errors [37]. Hereby, proposals arose for the quantitation of vitamin B_6_ in biological samples [38, 39], infant formula [40, 41], nutritional formula [42], and food [43–45]. Foodstuffs analyzed via LC-MS/MS comprise maize flour and green and golden kiwi as well as tomato pulp [43], Italian pasta [44], and beverages as well as dietary supplements [45].

So far, all studies revolving around LC-MS/MS analysis of foodstuff focus on simultaneous multivitamin analysis including some of the B_6_ vitamers and furthermore lack data on PNG. And [43] despite the rising implementation of LC-MS/MS into analytical chemistry with regard to the quantitation of vitamin B_6_, methods revolving around the simultaneous quantification of the individual vitamers in food utilizing SIDA remain still scarce. Hence, the aim of this study focused on the development of a LC-MS/MS method—including optimization of the sample workup and validation—capable of quantifying multiple vitamers of the B_6_ group in food samples [46, 47]. Hereby, special attention was given to PNG as a new substrate being included in an analytical method revolving around the analysis of vitamin B_6_.

## Materials and methods

### Chemicals and reagents

Pyridoxine hydrochloride (≥ 99%) was obtained from Alfa Aesar (Bellefonte, PA, USA) and pyridoxal hydrochloride (≥ 99.5%) from Alexis Biochemicals (Lausen, Switzerland). Pyridoxamine dihydrochloride (≥ 98%), pyridoxamine-5′-phosphate (≥ 98%), and pyridoxal-5′-phosphate monohydrate (≥ 97%) were purchased from Sigma-Aldrich (Bellefonte, PA, USA). 5′-*β*-Pyridoxine glycoside and the stable isotopically labeled standards [^13^C_3_]-PN, [^13^C_3_]-PL, and [^13^C_6_]-PNG were prepared in our laboratory, chromatographically purified, and characterized by NMR spectroscopy as published [48, 49]. Concentrations were determined by qNMR.

Formic acid (≥ 98%) was obtained from Sigma-Aldrich (Bellefonte, PA, USA), hydrochloric acid (32%) from VWR International GmbH (Darmstadt, Germany), and starch (high purity, order number: 101252) from Merck KGaA (Darmstadt, Germany). Acetonitrile (analytical grade), methanol (analytical grade), water (analytical and LC-MS grade), and isopropanol (LC-MS grade) were received from VWR International GmbH (Ismaning, Germany). Acetonitrile (LC-MS grade) was obtained from Carl Roth GmbH & Co., KG (Karlsruhe, Germany). Methanol (LC-MS grade) was purchased from Honeywell^™^ Riedel-de Häen^™^ (Seelze, Germany).

### Preparation of stock solutions and calibration standards

Stock solutions of the unlabeled and labeled vitamers were prepared in concentrations of 0.1, 1, and 10 μg/mL in water (LC-MS grade) and stored at − 28 °C in the dark. The stock solutions were stable for the length of the study (6 months) under these conditions.

### LC-MS/MS conditions

LC-MS/MS was carried out on a Shimadzu Nexera X2 UHPLC system (Shimadzu, Kyoto, Japan). A Shim-pack Velox PFPP column (2.1 × 100 mm, 2.7 μm, 90 Å, Shimadzu Corporation, Kyoto, Japan) protected by a Velox EXP Guard PFPP (2.1 × 5 mm, Shimadzu Corporation, Kyoto, Japan) was used as stationary phase and the temperature held at 30 °C. The flow rate was set to 0.3 mL/min. The binary gradient system comprised 0.1% formic acid in water (solvent A) and methanol (B). The mobile phase was held at 2% B for the first minute. The gradient raised linearly from 2 to 3.5% B during 1 min and remained at 3.5% B for 0.5 min. Afterwards, it raised to 5% B over the course of 0.5 min and was kept at 5% B for 4 min. Next, it raised to 50% B during 1 min and remained at 50% for 2 min. Finally, the mobile phase returned to 2% B over 1 min and the column was equilibrated for 3 min. The injection volume was 1 μL. As for the detection of the analytes, a triple quadrupole mass spectrometer (LCMS-8050, Shimadzu, Kyoto, Japan) was used that functioned in the positive electrospray ionization (ESI) mode for all analytes. Customization regarding the parameters of the interface proceeded as follows: nebulizing gas flow 3 L/min, heating gas flow 10 L/min, drying gas flow 10 L/min, heat block temperature 400 °C, interface temperature 300 °C, desolvation line temperature 250 °C, interface voltage 4 kV, and collision-induced dissociation gas pressure 270 kPa. MS/MS measurements were performed by the mass spectrometer in the scheduled multiple reaction monitoring (MRM) mode. The voltages for the fragmentation of the individual analytes were optimized by direct infusion of standard solutions of the vitamers at a concentration of 1 μg/mL. Confirmation of the analytes proceeded through two mass transitions, namely the quantifier and as qualifier. The mass transitions of the qualifiers and quantifiers of the analytes were checked for interfering matrix compounds from strawberry, banana, potato, and whole wheat flour extracts. Optimized voltages and collision energies, the retention time, and the quantifier/qualifier ratio of each analyte are listed in Table 1. Data acquisition was performed with LabSolutions software 5.80 (Shimadzu, Kyoto, Japan).

**Table 1:** Precursor ions and product ions of the labeled and unlabeled B_6_ vitamers, optimized fragmentation conditions, retention times, and quantifier/qualifier ratio

Analyte	Precursor ion *m/z*	Product ion *m/z*^a)^	Q1 pre-bias (V)	CE (V)	Q3 pre-bias (V)	Retention time (min)	Ratio quantifier/qualifier^b)^
PN	169.80	134.15	− 18	− 20	− 24	1.90	1.26
		152.15	− 14	− 14	− 36		
[^13^C_3_]-PN	172.80	137.15	− 18	− 20	− 24	1.88	1.18
		155.15	− 14	− 14	− 36		
PL	167.80	94.25	− 14	− 22	− 20	1.57	2.36
		150.20	− 14	− 13	− 16		
[^13^C_3_]-PL	170.80	95.25	− 14	− 22	− 20	1.57	2.50
		153.20	− 14	− 13	− 16		
PM	169.20	135.25	− 10	− 20	− 10	1.03	0.81
		152.25	− 10	− 14	− 30		
PMP	249.20	134.15	− 6	− 21	− 10	1.06	1.12
		232.10	− 18	− 13	− 12		
PNG	331.90	108.20	− 14	− 22	− 22	2.12	1.33
		152.20	− 14	− 23	− 18		
[^13^C_6_]-PNG	337.90	108.20	− 14	− 22	− 22	2.12	1.28
		152.20	− 14	− 23	− 18		

^a)^The first line reports the most intense MRM transition (quantifier) and the second line the second less intense one

^b)^Mean of five injections. The quantifier/qualifier ratios were determined in pure solvents

### Food samples

Fourteen food samples—among them five vegetables, eight fruits, and one flour sample (for detailed information about the origin of each sample, see Table S1 in the Electronic Supplementary Material, ESM)—were purchased from various supermarkets in Germany in single units (except for strawberry, where five units were combined and homogenized) and analyzed for their vitamin B_6_ content with the optimized and validated method. The foodstuff was stored in the fridge after purchase and prepared as fast as possible. Stems were removed. No food was peeled, except for banana and melon, where only the pulp was used. Seeds were removed in all samples except for strawberry.

### Optimization of the sample extraction

Generally, the blueprint for the sample workup followed a mild acidic extraction procedure including protein precipitation as well as centrifugation steps, subsequent reduction of the solvent, reconstitution of the sample, filtration, and measurement. Hereby, different aspects within the method optimization procedure such as sample weight and type as well as volume of extraction solvents, number and duration of extractions, and the composition of the uptake solvent were varied. All variations of the workup conditions used starch as matrix and were injected once after one single workup.

### Optimized sample preparation

One gram of a well-ground and homogenized (standard mixer equipped with blades) sample was weighed into a 50-mL centrifuge tube and the isotopically labeled internal standards were added to the sample (200 μL of [^13^C_3_]-PN (1 μg/mL), 100 μL of [^13^C_3_]-PL (1 μg/mL), and 200 μL of [^13^C_6_]-PNG (1 μg/mL, solvent: water (LC-MS grade)). Extraction proceeded through addition of 20 mL of 5% formic acid (v/v in H_2_O) and shaking the mixture horizontally (350 rpm, Kombi Schüttler KL2, Edmund Bühler GmbH) for 5 min. The sample was centrifuged (1790×*g*, Centrifuge 5810 R, Eppendorf AG) for 15 min at 4 °C and the supernatant transferred into a new 50-mL centrifuge tube. A total of 15 mL ACN was added to the supernatant in order to initiate protein precipitation and centrifuged again (1790×*g*, 15 min). Another 15 mL ACN was added and the centrifugation repeated. The supernatant was transferred in a round flask (100 mL), the solvent rotary evaporated under reduced pressure (Rotationsverdampfer Hei-Vap Value, Heidolph Instruments GmbH & Co. KG), and the residue reconstituted in 10 mL of a mixture of 0.1% formic acid in water and methanol (v/v = 1/1). The sample was membrane-filtered (0.22 mm, polyvinylidene fluoride, Macherey-Nagel GmbH & Co. KG) and analyzed by LC-MS/MS.

### Calibration and quantitation

PN, PL, and PNG were quantified using isotopically labeled internal standards, while PM and PMP were determined utilizing matrix-matched calibration in starch as matrix referring to isotopically labeled PN as internal standard. For PN and PL, the corresponding [^13^C_3_]-labeled compounds were used as internal standards, while PNG was analyzed with an [^13^C_6_]-labeled isotopologue.

Calibration functions of PN, PL, and PNG were established by mixing varying amounts of analyte (A) with constant amounts of internal standard (S). For PM and PMP, varying amounts of analyte (A) and constant amounts of internal standard (S) were spiked into vitamer-free starch and processed according to the procedure described above. The calibration was performed in molar ratios [*n*(A)/*n*(S)] between 0.01 and 100 (1:100, 1:75, 1:50, 1:10, 1:5, 1:2, 1:1, 2:1, 5:1, 10:1, 50:1, 100:1) for PN, PL, PM, and PNG and between 0.003 and 100 (1:300, 1:100, 1:50, 1:10, 1:5, 1:2, 1:1, 2:1, 5:1, 10:1, 50:1, 100:1) for PMP. The calibration functions were calculated from molar ratios [*n*(A)/*n*(S)] versus peak area ratios [*A*(A)/*A*(S)] after LC-MS/MS measurements using linear regression, which was verified using Mandel’s fitting test.

### Method validation

Determination of limits of detections (LODs) and limits of quantifications (LOQs) was performed as suggested by Vogelgesang and Hädrich [47] by spiking starch at four different spiking levels with PN (0.02, 0.1, 0.2, and 0.3 mg/kg), PL (0.07, 0.25, 0.5, and 0.7 mg/kg), PM (0.01, 0.035, 0.07, and 0.1 mg/kg), PMP (0.02, 0.08, 0.14, and 0.2 mg/kg), and PNG (0.01, 0.035, 0.07, and 0.1 mg/kg). Each level was worked up in triplicate and each extraction injected in triplicate. The extraction of vitamers and reduction of starch components were performed according to the procedure listed above followed by the measurement via LC-MS/MS.

The recoveries for each vitamer were determined by spiking the starch at three different spiking levels with PN (0.1, 0.2, and 0.3 mg/kg), PL (0.25, 0.5, and 0.7 mg/kg), PM (0.035, 0.07, and 0.1 mg/kg), PMP (0.08, 0.14, and 0.2 mg/kg), and PNG (0.035, 0.07, and 0.1 mg/kg), while each level was worked up in triplicate, and preparation according to the procedure described above followed by triple measurement via LC-MS/MS. The recovery rates of PN, PL, PM, PMP, and PNG were calculated as the ratio of detected and spiked amounts (*R* = found amount [mg/kg]/spiked amount [mg/kg]). Since the current study was of proof-of-principle and no reference material was available, recovery was taken for the assessment of trueness.

Strawberry, banana, potato, and whole wheat flour were used for the determination of precisions (calculated as relative standard deviations) by spiking the absent vitamers and preparation according to the procedure described above followed by measurement via LC-MS/MS. Hereby, 300 μL PL (1 μg/mL) was spiked to banana, potato, and whole wheat flour and 300 μL PM (1 μg/mL) to whole wheat flour. Inter-injection precisions were determined by spiking and working up one of each food samples and injecting (1 μL) it in quintuplicate into the LC-MS/MS instrument (*n* = 5). The intra-day precisions were calculated after spiking and working up one of each food samples in triplicate followed by injecting (1 μL) it triply into the LC-MS/MS, all in 1 day (*n* = 9). The inter-day precisions were determined by spiking and working up one of each food samples in triplicate followed by injecting (1 μL) it triply (first day) or twice (second and third day) into the LC-MS/MS, all on three separate days distributed over 3 weeks (*n* = 21).

## Results and discussion

This study revolved around the establishment of a method for the simultaneous quantification of the vitamers PN, PL, PM, PMP, and PNG in various food samples. The first steps started with the optimization of the sample workup and preparation. Next, the optimized method was validated, and lastly, various food samples were examined for their vitamin B_6_ content.

### Optimization of the sample workup

The development of a suitable method started with the optimization of the sample workup. All variations of the workup conditions were analyzed in a single determination using starch to scope a multitude of possibilities with regard to workup parameters. Hereby, either an explicit trend was obtained when investigating an individual workup method within one optimization step—where a definite variation of the workup conditions showed better results than other variations, e.g., utilization of 5% formic acid compared with pure water—or the outcome of all individual variations within one optimization step was similar (e.g., time of extraction). Traditional extraction procedures rely on the utilization of heat in an acidic environment and use this treatment to their favor in order to transform all forms of B_6_ into PN while focusing on the measurement of one vitamer within a multivitamin method. Because the present study focused on conserving the individual forms of B_6_, a milder procedure was pursued. Additionally, the utilization of mild agents for protein denaturation such as SSA was neglected due to the necessary separation prior to measurement, although they have been successfully applied in former studies revolving around HPLC analysis. Hence, this study’s goal with regard to the optimization focused on the establishment of a mild procedure including both the complete extraction of the individual vitamers and a mild process avoiding vitamer degradation.

**Fig. 2:**
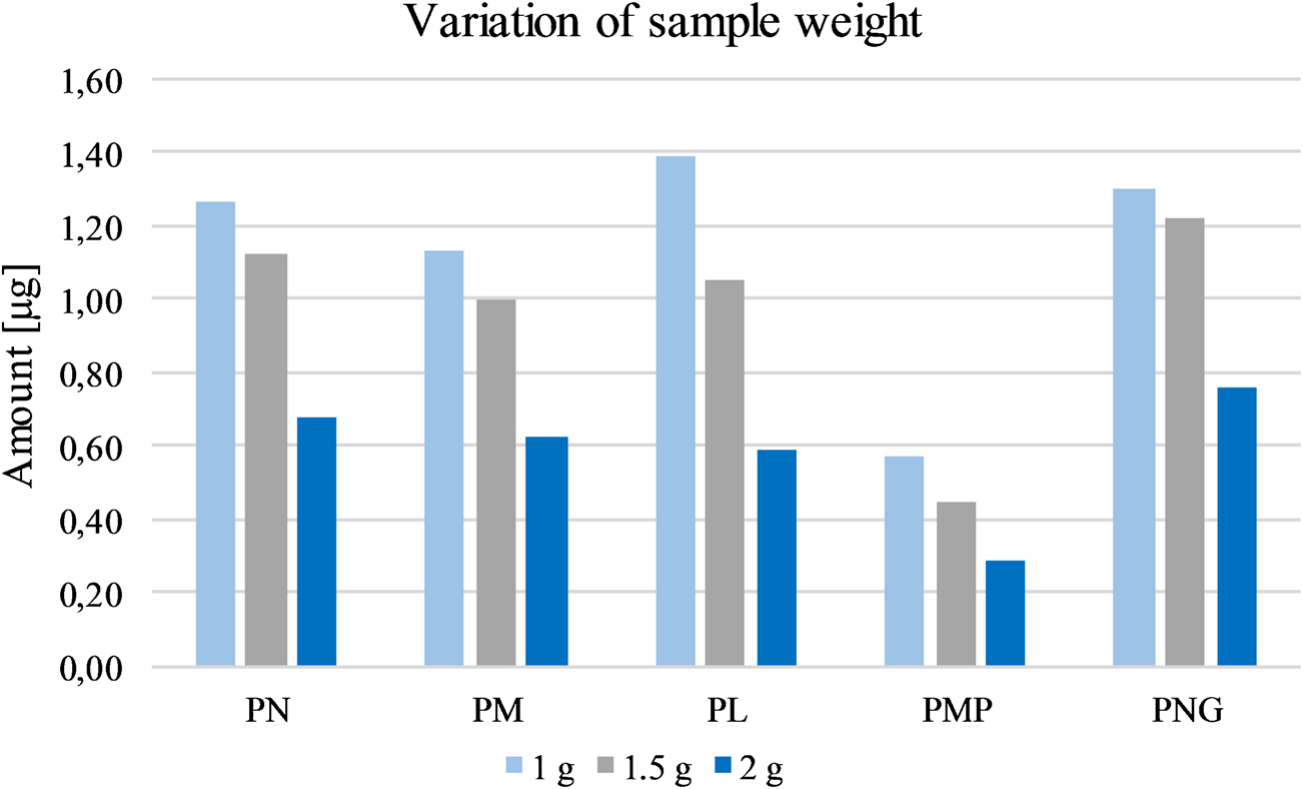
Optimization of the sample weight during workup using 1, 1.5, and 2 g starch as matrix. Each sample was determined once. Given amounts refer to the obtained quantity of the respective vitamer after workup

In a first survey, the weight of the test sample was optimized in order to assure a detectable amount of analyte and adequately reduce matrix effects. Thus, sample weights of 1.0, 1.5, and 2.0 g were examined. 1.0 g resulted in the best outcome, i.e., the highest specific concentration in the extract while worsening proportionally with increasing weight (Fig. 2).

When starting with 2.0 g, supernatants remained strongly turbid after the centrifugation, aggravating a separation of the matrix. Reported sample weights usually vary strongly depending on the analyzed matrix in the literature [27]. The sampling described with regard to food like whole wheat and refined wheat products [29, 50, 51], quinoa [34], potato, or various brans and soy flour [52] ranged roughly between 0.5 and 30 g.

The extraction of the B_6_ vitamers proceeded via acidic extraction and centrifugation procedures, since the application of cartridges for matrix reduction presents a difficult task due to the properties of the B_6_ group. Traditionally, sample workup involved the utilization of mineralic acids in combination with the application of heat, e.g., with the help of an autoclave, in order to release protein-bound B_6_ [53]. Depending on the reaction conditions, this procedure often also led to the degradation of phosphorylated and glycosylated derivatives and was combined with enzymatic methods to achieve a complete transformation of the vitamers into one substrate in order to analyze the total B_6_ content [19, 35, 54, 55]. Furthermore, milder procedures were applied for the determination of individual vitamers by addition of deproteinizing agents like perchloric acid, trichloroacetic acid (TCA), and sulfosalicylic acid (SSA) in order to keep the phosphorylated derivatives intact. These methods share the issue that the additive has to be removed before HPLC measurement [56, 57]. The establishment of a LC-MS/MS method including the application of internal standards in a SIDA allows the determination of the individual vitamers, thus making the hydrolysis and treatment with glycosidase dispensable.

**Fig. 3:**
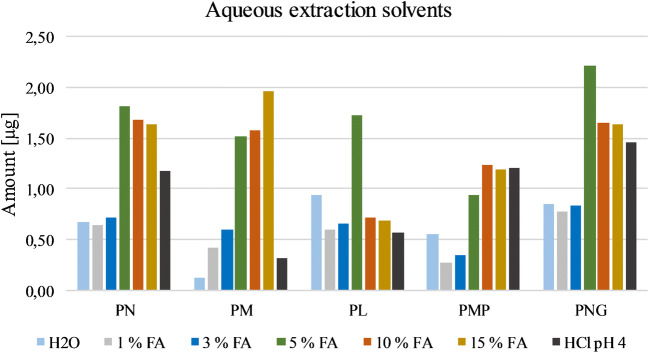
Testing various aqueous extraction solvents during sample workup. Given amounts refer to the obtained quantity of the respective vitamer after workup. FA, formic acid

In this study, extraction solvents consisting of various concentrations of formic acid (FA) in water (1, 3, 5, 10, and 15% vol in H_2_O) were studied (Fig. 3). Formic acid was chosen due to its simple incorporation into ESI-MS and overall increased stability of the vitamers in acidic environment. Furthermore, pure water and hydrochloric acid in water (pH = 4.0) were examined. Five percent FA showed on average the best results among the tested concentrations, since lower concentrations (1 and 3%) led to unsatisfying recoveries throughout all analytes and higher concentrations (10 and 15%) showed partially better (PM and PMP) or worse (PN, PL, and PNG) results. Hydrochloric acid was worse for the extraction of PM, while water showed overall bad extraction results for all vitamers. In the literature, hydrochloric or sulfuric acid is often used in combination with autoclaving to liberate the vitamers from the matrix. [19, 25–27]

In an additional set of experiments, mixtures of aqueous formic acid and organic solvents (acetonitrile/methanol) were tested as extraction media, but resulted in worse results compared with 5% FA in water.

**Fig. 4:**
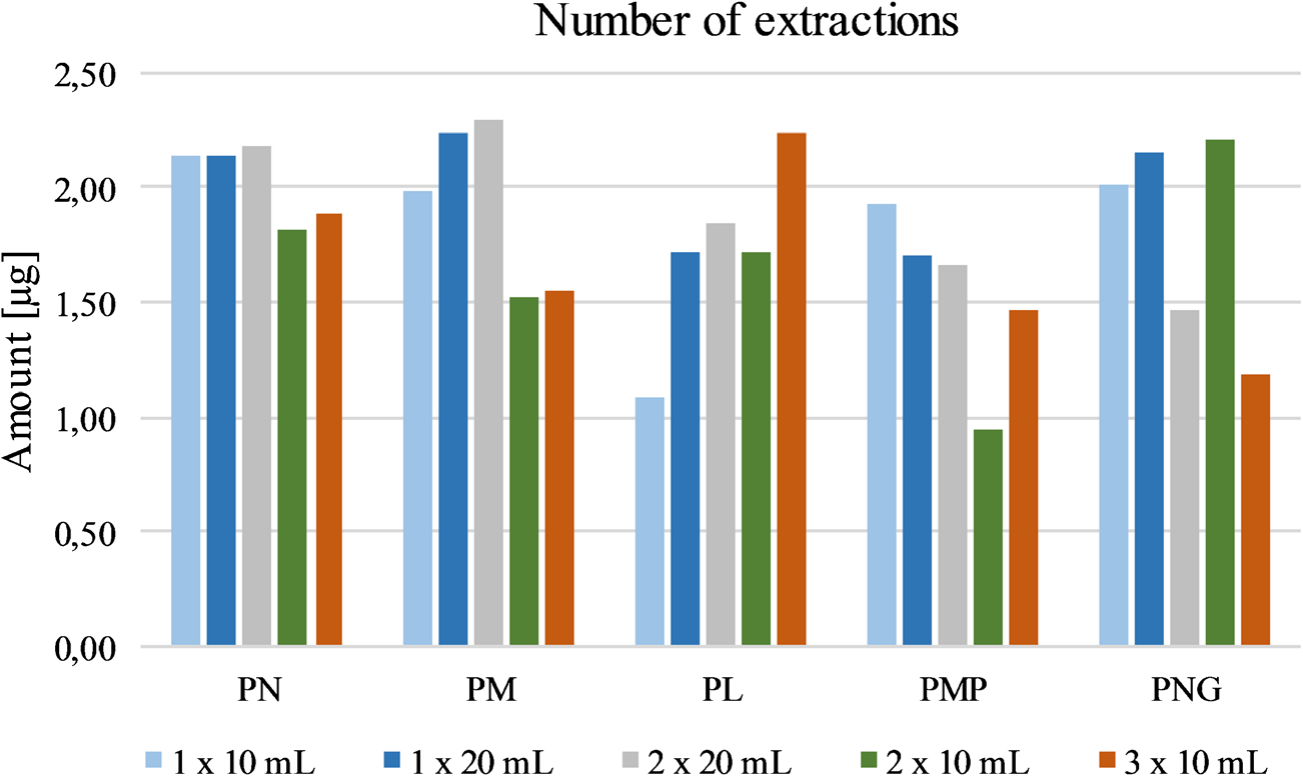
Testing the number of extractions with 5% formic acid. One extraction with 20 mL showed the best results for the B_6_ vitamers. Given amounts refer to the obtained quantity of the respective vitamer after workup

The next variations focused on the number and duration of extractions. Single (á 10 or 20 mL), double (á 10 or 20 mL), and triple extractions (á 10 mL) with 5% FA were preliminarily performed to find the optimal volume (Fig. 4). Hereby, extractions with 10 mL showed worse results compared with extractions with 20 mL. Single extractions with 10 mL showed bad results for PL, double extractions for PM and PMP, and triple extraction for PM and PNG. Extracting twice with 20 mL resulted in a lower recovery for PNG; hence, a single extraction with 20 mL solvent was used, delivering the best results compared with the other methods.

**Fig. 5:**
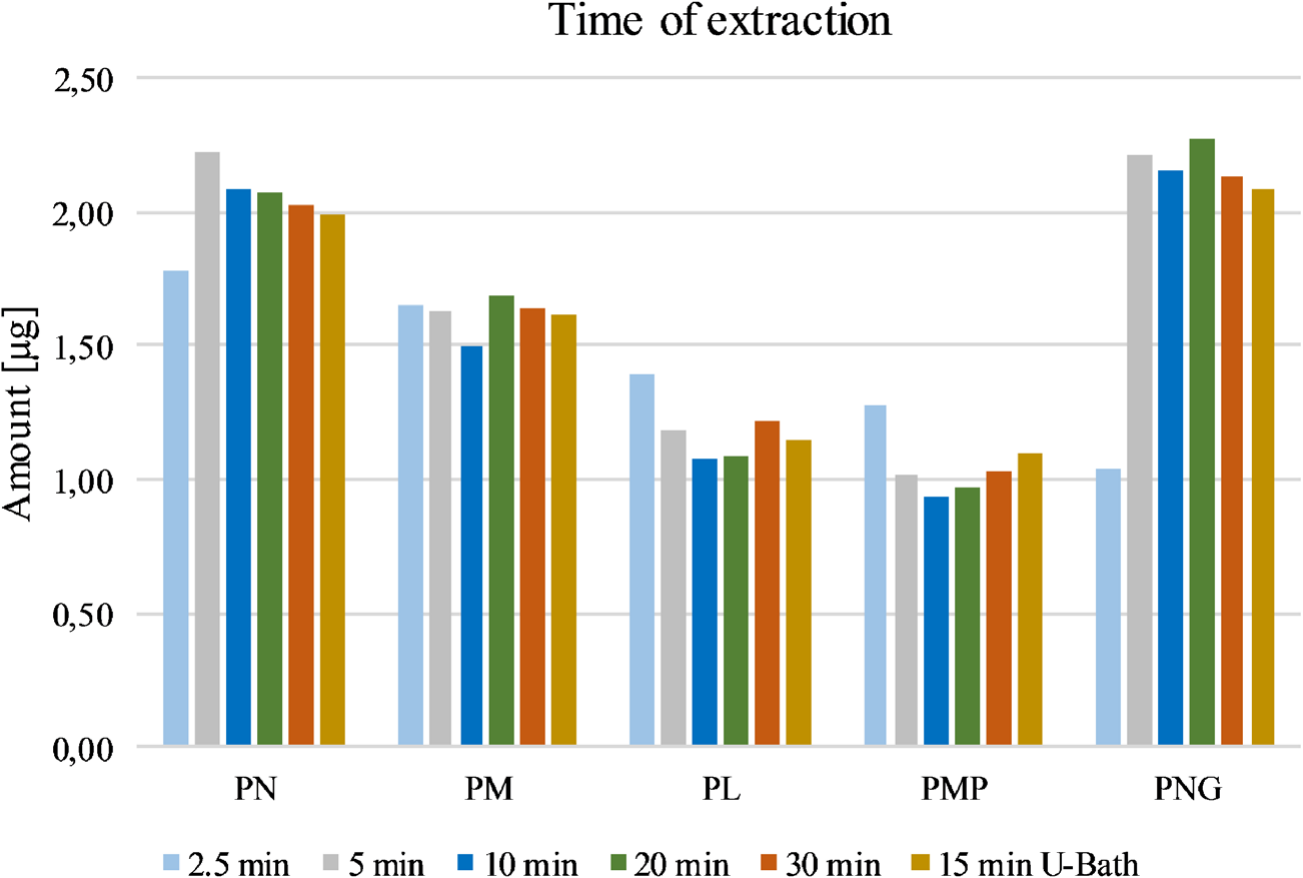
Testing the extraction time with 5% formic acid. An extraction time of 5 min showed best results for the vitamers. Given amounts refer to the obtained quantity of the respective vitamer after workup. U-bath: ultrasonic bath

With regard to the extraction time, not only different time frames (2.5, 5, 10, 20, and 30 min) were tested, but also the utilization of an ultrasonic bath (U-bath; 15 min) instead of a shaker (Fig. 5). Extraction times ranging from 5 to 30 min showed a similar outcome. 2.5 min showed slightly better results for PL and PMP, but worse for PN and PNG, making the method unsatisfying for the desired determination of all vitamers. Since a shorter extraction time generally is preferred, upcoming experiments were performed with an extraction time of 5 min.

Centrifugation is an important step in the separation of matrix components and finds its frequent application in the analytical quantification of vitamin B_6_ in methods using SSA extraction [58], while protein precipitation through addition of methanol or acetonitrile is seldom undertaken. In order to verify that the precipitate after centrifugation does not enclose any vitamer amounts, the residue was dissolved and checked via ESI-MS and no co-precipitated vitamers were detected. The separation of the proteins and the resulting increased sample volume required the removal of the solvent. Hence, the solvent was removed under reduced pressure at mild temperatures and the residue reconstituted with a defined volume.

**Fig. 6:**
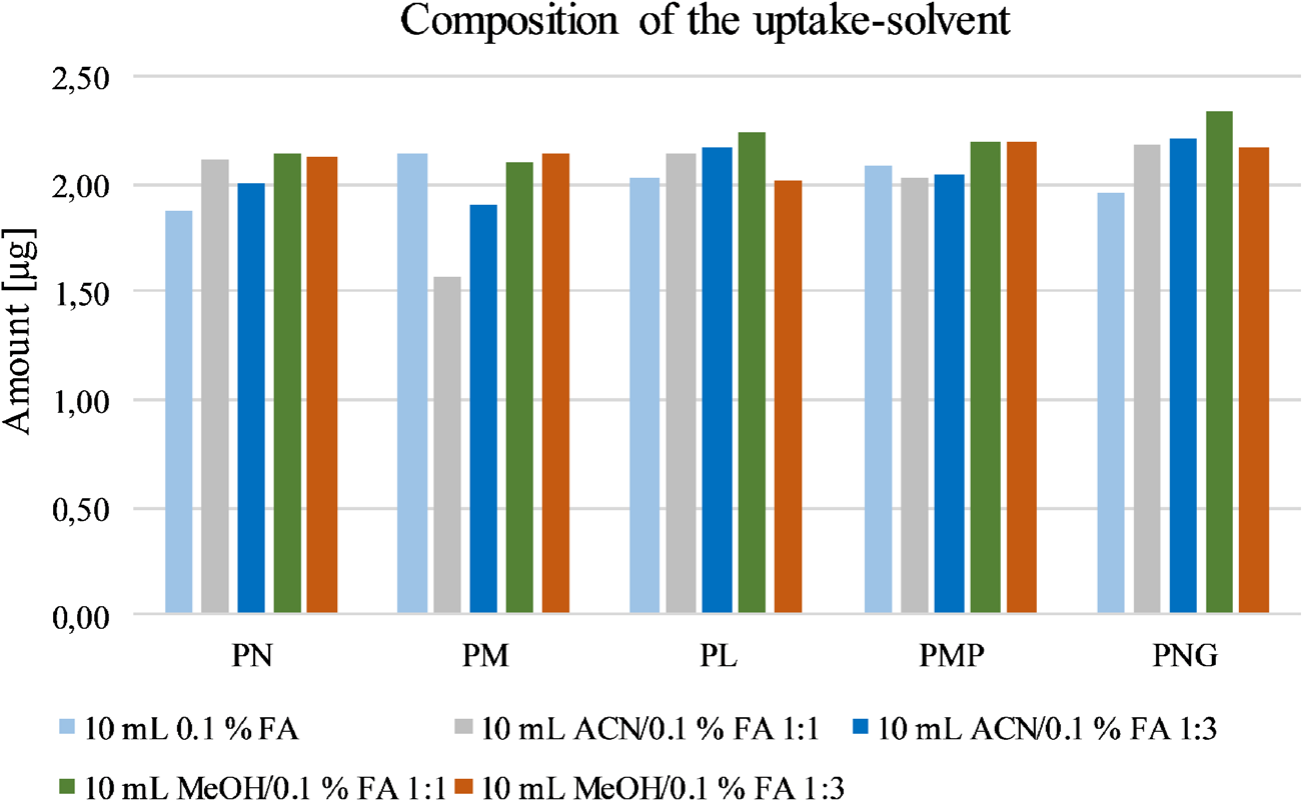
Testing the solvent for reconstitution of the extracts. A mixture of FA and methanol showed the best results as uptake volume. Given amounts refer to the obtained quantity of the respective vitamer after workup

As last part of the method development, the volume and composition of the uptake solvent were examined (Fig. 6). The residue was reconstituted with 1, 1.5, 5, or 10 mL of formic acid (0.1% vol in H_2_O). Hereby, 10 mL showed the best results in comparison with the other volumes.

Lastly, the composition of the uptake volume was optimized. Therefore, mixtures of 0.1% FA with ACN or MeOH (1:1 or 3:1, v/v) were used next to pure 0.1% FA. The different solvents showed similar results, but since the mixture of 0.1% FA in MeOH with a ratio of 1:1 (v/v) gave slightly better results, this composition was used as the uptake solvent [59].

### Optimization of LC-MS/MS conditions

Preliminary experiments revolved around the separation of the vitamers on different solid phases and suitable eluent composition. The comparison of C_8_, C_18_, biphenyl, biphenylpropyl, phenyl-hexyl, HILIC, and PFPP materials showed that all columns had issues with the separation of the vitamers (for detailed information about the columns and HPLC conditions, see Tables S2 and S3 in the ESM). Furthermore, PM and PMP always dwelled near the dead volume. Among the tested columns, the PFPP phase resulted in the best peak shape and shortest overall run time, thus allowing a higher sample throughput. The dead volume was measured with acetone to 0.90 min.

**Fig. 7:**
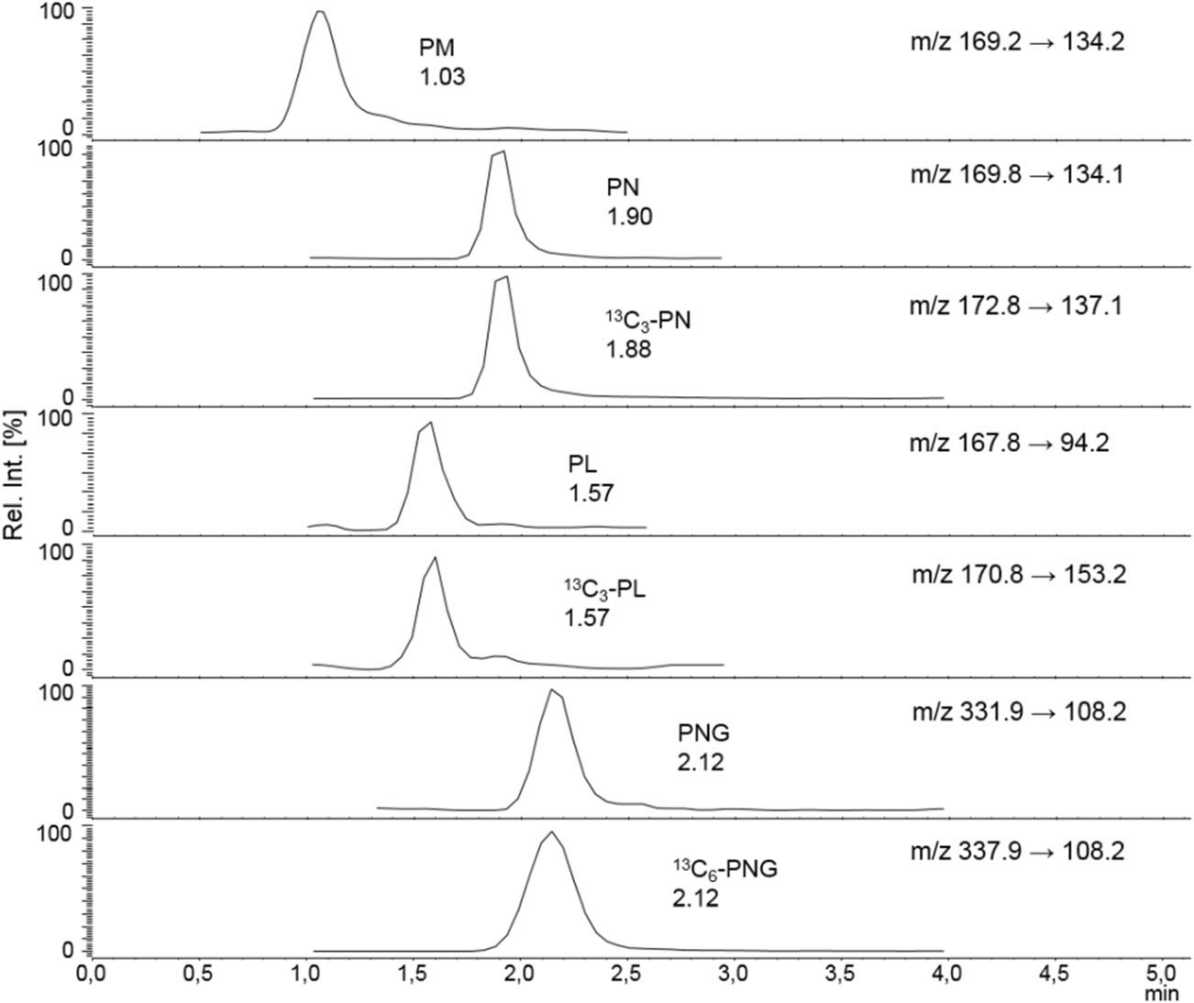
LC-ESI(+)-MS/MS chromatograms of an apple extract spiked with isotopically labeled standards measured with an Shim-pack Velox PFPP column (2.7 μm, 2.1 × 100 mm)

Since the B_6_ vitamers consist of different ionic forms depending on the pH of the eluent and show overall greater stability in acidic environment, various acid strengths were tested regarding their influence on the retention time while examining different mobile phases. Hence, mixtures of acetic acid (0.1% vol in H_2_O) and formic acid (0.01, 0.1, 0.5, and 1.0% vol in H_2_O) with acetonitrile/methanol were tested as eluents. For the HILIC columns, eluents consisting of ammonium formate buffers (5–10 mM) were used. In summary, only a binary gradient consisting of 0.1% formic acid in water and methanol resulted in the best separation and peak shapes. Figure 7 shows the LC-MS/MS chromatograms of an apple sample spiked with isotopically labeled standards in the positive ESI mode.

**Fig. 8:**
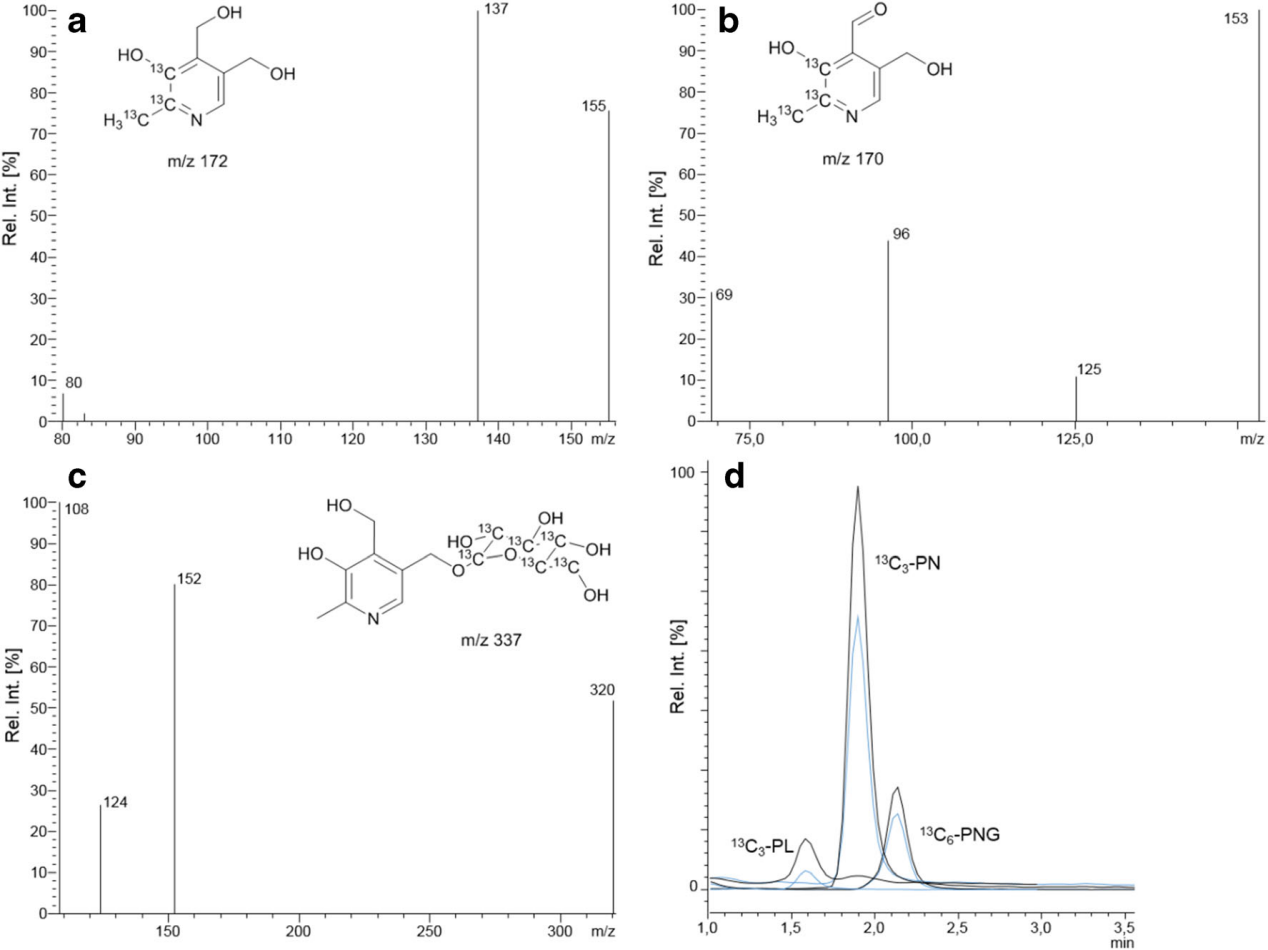
LC-ESI(+)-MS/MS spectra of [^13^C_3_]-PN (**a**, CE = − 20 V), [^13^C_3_]-PL (**b**, CE = 13 V), and [^13^C_6_]-PNG (**c**, CE = − 22 V). Chromatogram (**d**) depicts [^13^C_3_]-PN, [^13^C_3_]-PL, and [^13^C_6_]-PNG from a spiked watermelon extract. Black and blue mass transitions refer to the quantifier and qualifier (for *m*/*z* transitions, see Table 1) of the spiked standards, respectively

The B_6_ vitamers were measured in the positive ESI mode. Hereby, protonated molecules were used as precursor ions. Fragmentation patterns of the labeled standards resembled those of the respective unlabeled compounds (Fig. 8) [60–64].

Protonated [^13^C_3_]-PN (*m*/*z* 172.8) dissociated via loss of water (*m*/*z* 155.1), followed by an additional loss of water (*m*/*z* 137.1). The ESI-MS spectrum of protonated [^13^C_3_]-PL (*m*/*z* 170.8) also showed one loss of water (*m*/*z* 153.2), followed by other fragmentation steps [60]. Protonated [^13^C_6_]-PNG (*m*/*z* 337.9) fragmented via loss of glucose (*m*/*z* 152.2).

### Method validation

#### Calibration

The quantitation of the vitamers in this study was undertaken by using either the respective calibration function (for the vitamers PN, PL, and PNG) or the matrix-matched calibration function (in the case of PM and PMP with labeled PN as internal standard). An important part of the method development, therefore, involved the assessment of whether a linear or quadratic regression should be chosen for this analytical method. In this regard, the Mandel test presents an efficient method by comparing the residual standard deviation of the linear model with that of the nonlinear model [59]. In this study, the linearity of the calibration functions of PN, PL, PM, and PNG was confirmed for molar ratios [*n*(A)/*n*(IS)] from 0.01 to 100 and of PMP between 0.003 and 100 with correlation coefficients close to 1.000 (Table 2).

**Table 2:** Linear dynamic range, correlation coefficients (*R*^2^), limits of quantitation (LOQs), and limits of detection (LODs). Starch was used as matrix

Analyte	Molar ratio [*n*(A)/*n*(S)]	*a*^a)^	*R*^2^	LOD (mg/kg)	LOQ (mg/kg)
PN	0.01–100	1.24	0.999	0.018	0.054
PL	0.01–100	0.51	0.998	0.017	0.052
PM	0.01–100	2.87	0.999	0.0028	0.0085
PMP	0.003–100	1.38	0.998	0.02	0.059
PNG	0.01–100	0.89	1.000	0.01	0.031

^a)^Calibration fitting: *y* = *ax* + b

#### LODs and LOQs

LODs and LOQs of the SIDAs were calculated according to Vogelgesang and Hädrich [47] and ranged from 0.0028 to 0.02 mg/kg (LOD) and from 0.0085 to 0.059 mg/kg (LOQ) (Table 2). Hereby, the set limit of 10% regarding the variation coefficients was fulfilled, except of PMP, where a coefficient of 21% was obtained when determining the LOD.

LODs and LOQs of B_6_ were reported for various food samples [26], e.g., Italian pasta [44], corn steep liquor [65], sea food [24, 66], cow milk [23], maize flour [43], green or golden kiwis [43], tomato pulp [43], cooked sausage [28], and vary between 0.000016 and 0.043 mg/kg (LOD) and between 0.000033 and 0.129 mg/kg (LOQ) depending on the vitamer and matrix. Data specifically regarding PNG are scarce in the literature because the vitamer is commonly determined as PN after enzymatic treatment. Gregory et al. noted a LOQ of 0.02 mg/kg and LOD of 0.0001 mg/kg in various foodstuffs utilizing HPLC in combination with fluorescence detection [52, 58].

In a survey regarding the separation of various substrates—among others PN, PL, and PLP—on different HILIC stationary phases, LOQs of 0.006 to 0.03 mg/kg for PN and 0.019 to 0.068 mg/kg for PL were determined using stock solutions [67].

The obtained LODs and LOQs aligned well with the respective range reported in the literature. A comparison with methods using LC-MS/MS shows that the results obtained in this study by using starch as matrix align well with the data of Leporati et al. using Italian pasta made from wheat [44]. The latter authors obtained LODs between 0.001 and 0.003 mg/kg and LOQs between 0.003 and 0.01 mg/kg according to a signal-to-noise ratio (*S*/*N* = 3 and 10). In comparison with that, Gentili et al. found for maize flour LODs ranging from 0.009 to 0.0144 mg/kg and LOQs ranging from 0.027 to 0.0432 mg/kg [43].

Since one aim of this study lied in the elaboration of the first LC-MS/MS method to detect and quantify PNG next to several B_6_ vitamers, this study granted data for a first general comparison of LODs and LOQs with other methods often chosen for the detection of B_6_ in foodstuff (e.g., fluorescence detection).

#### Recoveries

The recoveries of the SIDAs and matrix-matched calibrations were determined at three different levels by spiking vitamer-free starch with PN, PL, PM, PMP, and PNG. Hereby, the targeted range between 70 and 120% was met with values ranging between 92 and 111% for PN, PL, and PNG, which were determined by SIDA, and between 98 and 104% for PM and PMP, whose measurement was done via matrix-matched calibration. Both procedures had relative standard deviations (RSDs) below 9% (Table 3).

**Table 3:** Recoveries of the single vitamers in starch

Analyte	Recovery (%)		
	Level 1	Level 2	Level 3
PN	109 ± 6	111 ± 4	103 ± 4
PL	100 ± 3	98 ± 3	99 ± 1
PM	98 ± 2	102 ± 1	98 ± 1
PMP	104 ± 6	99 ± 3	100 ± 3
PNG	92 ± 3	99 ± 8	95 ± 9

Recoveries described in various foods [24, 26], e.g., carrot [32], anchovy [32], garlic [32], chinoa [32], kiwi [43], tomato pulp [43], fish [66], cooked sausages [28], cow milk [23, 55, 68], Italian pasta [44], wheat [29], maize flour [43], and whole wheat flour [27], ranged between 15 and 118% depending on the matrix, extraction method, detection apparatus, and vitamer. Important factors when assessing the recoveries of B_6_ molecules refer to the extraction procedure and sample workup, since incomplete extraction or dephosphorylation/deglycosylation by enzymes has a crucial impact on the method. Differences in matrix composition play an additional role, thus complicating the determination. While utilizing 5% metaphosphoric acid during sample workup Sampson et al. measured recoveries between 57 and 101% of B_6_ vitamers in wheat, while Gentili et al. noted recoveries ranging from 40 to 96% in maize flour after matrix separation with C_18_ sorbent [29, 43]. Both did not include data on PNG. Thi Viet Do et al. received recoveries of 58 and 74% for PNG in carrot and garlic, respectively [32].

The recoveries measured during this study fit the expected values for a SIDA and matrix-matched calibration well. Losses during the workup were compensated for via reference to an internal standard. A comparison with literature data shows that the recoveries reported in this study with regard to similar food matrices (e.g., foods rich in starch) were better. This aspect can be traced back to the mild sample workup procedure in combination with application of SIDA during this study.

#### Precision

Calculation of inter-injection (*n* = 5), intra-day (*n* = 9), and inter-day (*n* = 21) precisions proceeded through spiking the missing vitamers (PL and PM) to whole wheat flour, potato, strawberry, or banana, subsequent preparation of the samples according to the procedure described above, and lastly injection into the LC-MS/MS instrument (Table 4) [46].

**Table 4:** Inter-injection, intra-day, and inter-day precisions given as relative standard deviation (RSD) for whole wheat flour, potato, strawberry, and banana

Analyte	Precision (RSD) (%)		
	Inter-injection (*n* = 5)	Intra-day (*n* = 9)	Inter-day (*n* = 21)
Whole wheat flour			
PN	8	8	6
PL	5	6	6
PM	6	7	8
PMP	0.4	9	7
PNG	7	10	2
Potato			
PN	5	9	7
PL	-	-	-
PM	5	8	7
PMP	1	8	6
PNG	4	7	6
Strawberry			
PN	3	6	5
PL	9	9	10
PM	7	9	5
PMP	2	7	4
PNG	5	5	8
Banana			
PN	9	9	8
PL	3	4	4
PM	9	8	5
PMP	5	10	10
PNG	9	9	8

“-” not determined

The relative standard deviation of the inter-injection precision ranged from 1 to 8% for whole wheat flour, 1 to 5% for potato, 2 to 9% for strawberry, and 3 to 9% for banana. Data for intra-day precisions ranged from 6 to 10% for whole wheat flour, 7 to 9% for potato, 5 to 9% for strawberry, and 4 to 10% for banana. Inter-day precisions ranged from 2 to 8% for whole wheat flour, 6 to 7% for potato, 4 to 10% for strawberry, and 4 to 10% for banana. All values met the targeted limit of 10%.

Inter- and intra-day precisions for foodstuff reported in the literature vary between 2 and 12 and between 1 and 5% depending on the matrix, vitamer, and method, respectively [23, 24, 26, 28, 43, 58, 66, 68]. Hereby, Gentili et al. reported inter-day precisions < 12% for B_6_ vitamers in maize flour, while Kall et al. noted an internal reproducibility of 5.6% for PN in Graham flour [26]. Gregory et al. assessed precisions ranging from 2.5 to 5.0% using vitamer standard solutions, including PNG with 3.8% [58].

The precisions for PL in potato were not evaluable despite multiple measurements. Interestingly, precisions determined using whole wheat flour, which arguably can be seen as relatively similar with regard to the composition of constituents, were assessable. In general, PL—next to PLP—had, in accordance with the literature, the lowest intensity throughout this study when optimizing the method, which makes the analysis of the substrate vulnerable to factors such as matrix effects. Nevertheless, the implementation of whole wheat flour in this study allows the comparison with food samples rich in starch.

Overall, the good recoveries, the low relative standard deviations of the precision measurements, and LODs/LOQs coinciding with the literature affirmed the development of a robust method. Hence, sample workup/preparation and the LC-MS/MS method present a suitable tool for the analysis of plant-based food such as fruits, vegetables, and cereals.

### Analysis of vegetables, fruits, and cereals

For the analysis of plant-based food, various vegetables (*n* = 5), fruits (*n* = 8), and a flour sample (*n* = 1) were purchased from local super markets and quantitatively analyzed for their content of the B_6_ vitamers PN, PL, PM, PMP, and PNG (Table 5).

**Table 5:** Contents of vitamin B_6_ vitamers in diverse food samples

Food	PN	SD	PL	SD	PM	SD	PMP	SD	PNG	SD
Broccoli	0.042	2.2	0.075	0.04	0.116	0.03	0.021	2.5	0.053	6.7
Cauliflower	0.047	3.5	0.075	14.5	0.002	4.0	0.019	3.9	0.019	3.2
Carrot	0.006	5.1	0.006	4.0	< LOD	-	0.019	4.5	0.157	9.8
Green pepper	0.005	4.0	0.031	8.9	0.110	19.8	0.019	1.0	0.092	9.3
Potato	0.015	9.2	< LOD	-	0.030	8.2	0.030	8.2	0.305	7.2
Whole wheat flour	0.034	8.1	< LOD	-	< LOD	-	0.016	9.1	0.134	9.7
Strawberry	0.004	6.3	0.078	9.0	0.007	6.5	0.008	6.5	0.008	5.2
Banana	0.105	8.8	< LOD	-	0.043	10.0	0.086	10.0	0.027	8.7
Nectarine	0.008	2.4	0.006	3.2	0.164	0.03	< LOD	-	0.005	3.0
Apricot	0.031	6.0	0.007	0.8	0.155	10.9	< LOD	-	0.019	4.8
Galia melon	0.002	1.1	0.003	3.0	< LOD	-	< LOD	-	0.027	8.0
Watermelon	0.006	1.5	0.006	4.6	0.008	10.1	0.019	6.5	0.082	11.1
Apple	0.018	5.0	0.003	0.8	0.002	10.9	< LOD	-	0.005	3.6
Orange	0.004	0.8	< LOD	-	0.039	8.2	0.020	8.2	0.082	14.2

Data are expressed as mg/100 g food sample and are given as means ± standard deviation (%). Values are means of two analytical replicates, which each were injected twice

#### Content of B_6_ in vegetables, fruits, and cereal

A first glance at the data reveals that all five vitamers were detected in broccoli, cauliflower, green pepper, strawberry, and watermelon. While green pepper (0.11 mg/100 g), nectarine (0.164 mg/100 g), and apricot (0.155 mg/100 g) stood out through high amounts of PM, cauliflower (0.075 mg/100 g), and strawberry (0.078 mg/100 g) contained large quantities of PL. Broccoli included large quantities of both PM (0.116 mg/100 g) and PL (0.075 mg/100 g). The highest content of PMP (0.086 mg/100 g) was found in banana. By contrast, watermelon surpassed other foods with regard to PNG (0.082 mg/100 g) in comparison with the other vitamers.

Although the numerous representations of B_6_ vitamers in broccoli being in accordance with the literature, elucidated data on the individual compounds vary significantly. While Gregory et al., Kabir et al., and Bognar and Ollilainen reported low quantities of PN (0.032–0.036 mg/100 g), PM (0.002–0.028 mg/100 g), PMP (0.041 mg/100 g), and PL (0.014–0.037 mg/100 g) after extraction with sulfosalicylic acid or autoclaving with 0.10 M HCl at 120 °C, the amount of PNG, on the one hand, was comparatively high (0.078–0.186 mg/100 g) [27, 58, 69]. Kall et al. also utilizing autoclaves for extraction purposes, on the other hand, noted high amounts of PL (0.118 mg/100 g) in comparison with the other vitamers (0.028–0.068 mg/100 g), taking treatment with phosphatases into calculation [26].

The comparatively high contents of PL (0.075 mg/100 g) and PN (0.047 mg/100 g) in cauliflower were in accordance with reported proportions (PL: 0.121 mg/100 g, PN: 0.052 mg/100 g) [26]. Although a higher quantity of PMP (0.019 mg/100 g) compared with PM (0.002 mg/100 g) was found in this study, the overall amount of the amine derivative of the B_6_ group matches with the literature (PM: 0.02 mg/100 g), considering the utilization of phosphatase during sample workup [26]. Comparing the data on raw cauliflower with reported results, the lower amount of PNG (0.019 mg/100 g) compared with those of the other vitamers is adequate.

Inspired by being an exceptional source of folates [70] and since no data on the individual content of B_6_ was reported in the literature, strawberries were implemented in this study. Hereby, vitamin B_6_ was strongly present as PL (0.078 mg/100 g) compared with the other vitamers (0.004–0.008 mg/100 g).

Kall et al. published data regarding PN (0.023 mg/100 g), PL (0.008 mg/100 g), and PM (0.005 mg/100 g) in apple after sample workup including an acid hydrolysis step (0.1 M HCl) in an autoclave [26]. The results for PN (0.018 mg/100 g), PL (0.003 mg/100 g), and PM (0.002 mg/100 g) from this study fit accurately with respect to the proportion of the vitamers. Furthermore, 0.005 mg/100 g PNG were detected with the presented method.

In this study, a high proportion of vitamin B_6_ in green pepper was present as PM (0.11 mg/100 g) and PNG (0.092 mg/100 g), while the other vitamers were detectable only in lower amounts (0.005–0.031 mg/100 g). Nishimura et al. examined red pepper (Japanese tougarashi) and converted all vitamers into the highly fluorescent 4-pyridoxolactone by enzymatic processes and acidic hydrolysis. Hereby, the large quantities of PN (0.91 mg/100 g) and PNP/PNG (0.546 mg/100 g, not differentiated) surpassed the remaining vitamers PL (0.089 mg/100 g), PM (0.067 mg/100 g), and PMP (0.242 mg/100 g) [25]. These values appear much higher than those measured by our method; however, lack of representativeness and biological variations have to be kept in mind and point to the fact that our study was just for proof-of-principle.

Literature data on the content of individual vitamers of B_6_ in oranges is not available. In orange juice, values on PNG (0.198 mg/100 g) exceeded the other vitamers (0.004–0.026 mg/100 g), indicating that the glycosylated form amounts up to 92% of the B_6_ content [58]. The orange analyzed in this study also contained comparatively high amounts of PNG (0.082 mg/100 g) to the other vitamers (0.004–0.039 mg/100 g).

Banana was the only food sample in this study, in which B_6_ vitamers were mostly represented by PN (0.105 mg/100 g). Besides, PMP (0.086 mg/100 g) and PM (0.043 mg/100 g) were present in high amounts, while the glycosylated derivative (0.027 mg/100 g) was found in comparatively lower amounts. This general distribution is also depicted in the literature, where PN (0.104–0.389 mg/100 g) and PM (0.067–0.482 mg/100 g) exceed the other vitamers (0.007–0.042 mg/100 g) [26, 58, 71]. In addition, according to previous literature, the ratio between PM and PMP is mostly shifted towards the free molecule due to involvement of enzymatic processes during sample preparation. Quantities of PMP, if reported, hereby remain low (0.007 mg/100 g) [58]. Contrarily, the mild extraction used in this study resulted in a twofold amount of PMP in comparison with PM, resulting in a similar trend to cauliflower, where more phosphorylated substrate was found.

In order to gain insight into the complete content of B_6_ vitamers in foods, and therefore compare the total B_6_ content without enzymatic steps during sample workup, the present method should be expanded by PLP and possibly PNP. PLP was not included due to lack of internal standard. PNP is not commercially available and, therefore, making the preparation of an internal standard via a tedious synthesis necessary. Nevertheless, to the best of our knowledge, no data is available on this substrate, making it an interesting focus for future studies.

#### Content of PNG in food

PN and 5′-*β*-PNG were present in all 14 samples. More precisely, approximately equivalent amounts were found in broccoli (PN, PNG = 0.042 mg/100 g, 0.053 mg/100 g), strawberry (0.004 mg/100 g, 0.008 mg/100 g), nectarine (0.008 mg/100 g, 0.005 mg/100 g), and apricot (0.031 mg/100 g, 0.019 mg/100 g). More PN was quantified in cauliflower (0.047 mg/100 g, 0.019 mg/100 g), banana (0.105 mg/100 g, 0.027 mg/100 g), and apple (0.018 mg/100 g, 0.005 mg/100 g), whereas higher amounts of PNG were obtained in carrot (0.006 mg/100 g, 0.157 mg/100 g), green pepper (0.005 mg/100 g, 0.092 mg/100 g), potato (0.015 mg/100 g, 0.305 mg/100 g), whole wheat flour (0.034 mg/100 g, 0.134 mg/100 g), Galia melon (0.002 mg/100 g, 0.027 mg/100 g), watermelon (0.006 mg/100 g, 0.082 mg/100 g), and orange (0.004 mg/100 g, 0.082 mg/100 g). Since plants use glycosylation for storage purposes, the abundant distribution of PNG throughout all samples is comprehensible [58]. Many quantifications of glycosylated derivatives of B_6_ in the literature result from enzymatic treatment and HPLC/fluorescence analysis. To the best of our knowledge, no data is available on the determination of PNG via LC-MS/MS and SIDA, making this the first study of this sort.

Among the tested samples, PNG accounted for a major part of the B_6_ content in whole wheat flour, potato, and carrot. Whole wheat flour contained 0.134 mg/100 g PNG next to 0.034 mg/100 g PN and 0.016 mg/100 g PMP. This is in accordance with reported data, where PNG (0.014–0.149 mg/100 g) accounts for the major part of B_6_ in various flour samples, e.g., wheat, whole wheat, Graham, rye, oat, maize, all-purpose, and soy flour. PN (0.006–0.41 mg/100 g), PM (0.010–0.044 mg/100 g), and PMP (0.01–0.024 mg/100 g) were found in lower amounts [26, 27, 30, 31, 43, 50, 51, 72].

In potato, PNG (3.05 mg/kg) also contributed mainly to the overall B_6_ content compared with the other vitamers (0.015–0.03 mg/100 g). Values reported in the literature also contained higher proportions of PNG (0.1 mg/100 g) next to PN (0.024–0.195 mg/100 g), PM (0.014–0.05 mg/100 g), and PL (0.009–0.089 mg/100 g) [26, 27, 73]. Investigating the impact of storage on the content of vitamin B_6_ in potato, Addo and Augustin found 0.439 mg/100 g PN, 0.321 mg/100 g PM, and 0.301 mg/100 g PNG after extraction with SSA and HPLC analysis. Furthermore, storage over 9 months led to a fourfold increase of PN glycoside, which was probably related to adjustments of the osmotic pressure at the given storage temperature [57].

Next to cereals and potatoes, carrots were also associated with increased quantities of PNG. Utilizing PNG isolated from alfalfa seeds and fluorescence detection, Gregory et al., on the one hand, determined 0.145 mg/100 g and 0.606 mg/100 g PNG in raw carrots [52, 58]. Thi Viet Do et al., on the other hand, chose the approach of converting all vitamers into 4-pyridoxolactone through enzymatic procedures and reported 0.242 mg/100 g [32]. In this survey, PNG (0.157 mg/100 g) constituted the highest represented vitamer, as well. The residual B_6_ molecules accounted for 0.006 to 0.019 mg/100 g, which aligns well with reported results (0.003–0.064 mg/100 g) [32, 52, 58].

In summary, the data regarding the commonly examined vitamers PN, PL, PM, and the phosphates thereof found during this study fit well with the literature values, affirming the validity of the developed method. Furthermore, utilizing LC-MS/MS and SIDA, more insight into the individual PNG content of plant-based food was presented. Here, the data aligns with reports from methods using enzymatic treatment and fluorescence detection.

## Concluding remarks

LC-MS/MS in combination with SIDA presents a powerful tool for the determination of individual B_6_ vitamers in foodstuff. This study revolved around the development of a LC-MS/MS method including the optimization of the sample workup, the validation of the method validated, and lastly, its application in the analysis of various food samples. For the first time, the individual content of PNG in food was determined with ESI-MS/MS without enzymatic steps or divergent internal standards. Future applications of this method allow the accurate investigation of nutritional aspects of this group of vitamins such as effects of processing and bioavailability.

## Electronic supplementary material

ESM 1 (PDF 393 kb)

## Abbreviations

CE: Collision energy
PN: Pyridoxine
PL: Pyridoxal
PM: Pyridoxamine
PLP: Pyridoxal phosphate
PMP: Pyridoxamine phosphate
PNP: Pyridoxine phosphate
PNG: Pyridoxine glycoside
SSA: Sulfosalicylic acid
