# Supplementary material for: Development of a LC-MS/MS method using stable isotope dilution for the quantification of individual B6 vitamers in fruits, vegetables, and cereals
Source: Anal Bioanal Chem. 2020 Aug 14;412(26):7237–52. doi: 10.1007/s00216-020-02857-5 (PMC7497486; doi:10.1007/s00216-020-02857-5)
Supplement: Supplementary file 1 — (PDF 393 kb) [file 216_2020_2857_MOESM1_ESM.pdf]

Analytical and Bioanalytical Chemistry

Electronic Supplementary Material

Development of a LC-MS/MS method using stable isotope dilution for the quantification of individual B_6 vitamers in fruits, vegetables, and cereals

Thomas Bachmann, Andrea Maurer, Michael Rychlik

1. Sample Details

Table S1 Food samples and their origin

food	origin
strawberry	Sonner Strawberry plantation, Germany
banana	Global Fruit Point GmbH, Buxtehude, Germany
potato	Agropa Handels GmbH, Brunnen, Germany
whole wheat flour	Rosenmühle GmbH, Landshut, Germany
watermelon	SanLucar Fruit S.L., Valencia, Spain
galia melon	SanLucar Fruit S.L., Valencia, Spain
nectarine	SanLucar Fruit S.L., Valencia, Spain
cauliflower	Erzeugergroßmarkt Langförden-Oldenburg eG, Vechta-Langförden, Germany
broccoli	Erzeugergroßmarkt Langförden-Oldenburg eG, Vechta-Langförden, Germany
apple	Erzeugergroßmarkt Langförden-Oldenburg eG, Vechta-Langförden, Germany
carrot	Klaus Kaufmann, Freisbach, Germany
orange	SanLucar Fruit S.L., Valencia, Spain
apricot	Soursos S. A., Argos, Greece
green pepper	Erzeugergroßmarkt Langförden-Oldenburg eG, Vechta-Langförden, Germany

2. Columns and conditions used for method optimization

Table S2 Columns used during the method optimization

column	manufacturer
Synergi 4u Fusion RP 80 Å, 250 x 3,0 mm, 4 micron	Phenomenex, Aschaffenburg, Germany
Synergi 4u Polar RP 80 Å, 150 x 2,0 mm, 4 micron	Phenomenex, Aschaffenburg, Germany
Synergi 4u Hydro RP 80 Å, 250 x 3,0 mm, 4 micron	Phenomenex, Aschaffenburg, Germany
EC 250/3 Nucleosil 100-5 C18	Macherey-Nagel, Düren, Germany
Nucleosil 5u C8 100 Å, 250 x 4,6 mm, 5 micron	Phenomenex, Aschaffenburg, Germany
Jupiter 5u C18 300 Å, 250 x 4,6 mm, 5 micron	Phenomenex, Aschaffenburg, Germany
EC 250/4,6 Nucleodur π^2, 5 µm	Macherey-Nagel, Düren, Germany
EC 250 /4,6 Nucleodur HILIC, 5 µm	Macherey-Nagel, Düren, Germany
Kinetex 2.6u Biphenyl 100 Å, 150 x 4,6 mm	Phenomenex, Aschaffenburg, Germany
Luna 5u Phenyl-Hexyl 250 x 4,6 mm, 5 micron	Phenomenex, Aschaffenburg, Germany

Table S3 Columns and HPLC conditions tested during method optimization

Column	Eluent A	Eluent B	Flow
Synergie Fusion RP (C18)	0.1 % Formic acid in water (95 %)	Methanol (5 %)	0.6 mL/min
Synergi Polar RP (Phenyl)	0.1 % Formic acid in water (90 %)	Methanol (10 %)	0.4 mL/min
Synergi Hydro RP (C18)	0.1 % Formic acid in water (gradient)	Acetonitrile (gradient)	0.4 mL/min
Nucleosil C18	0.1 % Formic acid in water (95 %)	Methanol (5 %)	0.4 mL/min
Nucleosil C8	0.1 % Formic acid in water (95 %)	Methanol (5 %)	0.8 mL/min
Jupiter C18	0.1 % Formic acid in water (90 %)	Methanol (10 %)	0.8 mL/min
Nucleodur π^2 (Biphenylpropyl)	0.1 % Formic acid in water (100 %)	-	0.6 mL/min
Nucleodur HILIC	10 mM Ammonium formate pH 4.6 (50 %)	10 mM Ammonium formate pH 4.6/Acetonitrile 1:9 (50 %)	0.8 mL/min
Kinetex Biphenyl	0.1 % Formic acid in water (95 %)	Methanol (5 %)	0.4 mL/min
Luna Phenyl-Hexyl	0.1 % Formic acid in water (95 %)	Methanol (5%)	0.8 mL/min
Nucleodur HILIC	5 mM Ammonium formate pH 4.6 (60 %)	5 mM Ammonium formiate pH 4.6/Acetonitrile 1:9 (40 %)	0.4 mL/min
